# Supplementary material for: Unbreakable distributed storage with quantum key distribution network and password-authenticated secret sharing
Source: Sci Rep. 2016 Jul 1;6:28988. doi: 10.1038/srep28988 (PMC4929494; doi:10.1038/srep28988)
Supplement: Supplementary Information [file srep28988-s1.pdf]

Supplementary information

Manuscript title:

Unbreakable distributed storage with quantum key distribution network and password-authenticated secret sharing

Author list:

M. Fujiwara, A. Waseda, R. Nojima, S. Moriai, W. Ogata & M. Sasaki

Sketch of security proof of password-authenticated secret sharing

The scheme has the following three properties.

Theorem 1 If t corrupted storage servers try together to change the reconstructed data by deviating from the protocol, the data owner can detect it with probability of $(1 - l/q)$ if password P is randomly chosen.

(Proof) Manipulating 0's shares sent to honest servers in **Pre-computation and communication phase** and the responses sent back to the data owner in **Data Reconstruction phase,** t corrupted storage servers make the data owner reconstruct forged data blocks $\tilde{F}_i \neq F_i(0)$. The probability that *MAC* of forged data blocks $(\tilde{F}_l, \ldots, \tilde{F}_1)$ equals to $\tilde{F}_{l+1}$ is l/q, because *MAC* is calculated by using an l-degree polynomial that has (at most) l solutions. This means that such malicious behavior can be detected with probability of $(1 - l/q)$.

Theorem 2 The total information which t corrupted storage servers can see in the protocol is independent from the data owner's password p and stored secret data D, if the other servers and the data owner follow the protocol.

(Proof) We consider an attacker who corrupts t servers. The information that the attacker can see in **Registration phase** is t shares of password P and secret data D, which are generated by using t-degree polynomials. Therefore, they are independent from P and D as in Shamir's SS. The information that the attacker can see in **Pre-computation and communication phase** is t shares of random numbers and "0" computed by honest servers, which are clearly independent from P and D.

The information that the attacker can see in **Data reconstruction phase** is t shares of P'. Even if $P' = P$, the shares are independent from P and D.

Note that this situation does not change even if the corrupted servers send fake shares to honest servers.

Theorem 3 Even if an attacker first corrupts t of storage servers, then participates in Data reconstruction phase pretending to be a data owner by utilizing the corrupted servers, the total information which the attacker can obtain is no information other than whether the guessed password P' is equal to the correct password P or not.

(Proof) Here we describe the proof in the case where there is only one data block. The

generalization to the case of multiple data blocks is straightforward.

We consider an attacker who corrupts at most t servers $C = \{c_1, \dots, c_t\} \subseteq \{1, \dots, n\}$ and tries to get some information on password P and/or secret data D by pretending the data owner in **Data reconstruction phase**. In **Data reconstruction phase**, the attacker chooses a set of $2t + 1$ servers, L, in which all corrupted servers are included. Then, $\widetilde{H} = \{1, \dots, n\} \setminus C$ is the set of honest servers, $H = L \cap \widetilde{H} = \{h_1, \dots, h_{t+1}\}$ is the set of the honest servers that join the request-response process. The attacker can obtain all information in the corrupted servers and all information what those servers can observe in all phases. Furthermore, the attacker and the corrupted servers C can generate messages in arbitrary way and send them to honest servers $\widetilde{H}$. Without loss of generality, we assume $c_1 < \cdots < c_t$ and $h_1 < \cdots < h_{t+1}$.

First, we list up all information the attacker views. In **Registration phase**, the attacker obtains the shares of P and D generated by the data owner using random polynomials f_{P} and f_{D} of degrees at most t and $2t$, respectively. In each **Pre-computation and communication phase**, the attacker obtains the shares of a random number R_h and "0" generated by honest server h using random polynomials f_{R_h} and f_{0_h} of degrees at most t and $2t$, respectively. In each **Data reconstruction phase (3-4)**, the attacker obtains F_h as response which is computed by honest server h according to the scheme procedure. Thus, the information the attacker views through this attack is as follows:

$$V_1 = \{f_{\mathrm{P}}(c) \mid c \in C\},$$
$$V_2 = \{f_{\mathrm{D}}(c) \mid c \in C\},$$
$$V_3 = \{f_{\mathrm{R}_h}(c) \mid h \in H, c \in C\},\ V'_3 = \{f_{\mathrm{R}_h}(c) \mid h \in \widetilde{H} \setminus H, c \in C\},$$
$$V_4 = \{f_{0_h}(c) \mid h \in H, c \in C\},\ V'_4 = \{f_{0_h}(c) \mid h \in \widetilde{H} \setminus H, c \in C\},$$
$$V_5 = \{F_h \mid h \in H\}.$$

Clearly, V_1, V_2 themselves do not leak any information about password P and secret data D. Also, V'_3, V'_4 give no information to the attacker, because they are not used in **Data reconstruction phase**. On the other hand, V_3 and V_4 have the possibility to leak some additional information because they are related to the response V_5 through f_{R_h} and f_{0_h}. So, we consider (V_3, V_4, V_5).

Let $R_c^{(h)}$ and $Z_c^{(h)}$ be the values sent from corrupted server c to honest server h as a share of random number and "0", respectively, in **Pre-computation and communication phase**. Let $P'^{(h)}$ be the value that the attacker, at the beginning of **Data reconstruction phase,** sends to honest server h as a request. Note that these values may not be determined from polynomials, but there exists a unique polynomial $f_{\mathrm{P}'}(x)$ of degree t which satisfies $f_{\mathrm{P}'}(h) = P'^{(h)}$ for all $h \in H$. Hereafter, we show that V_3, V_4, V_5 has no additional information, unless $P \neq f_{\mathrm{P}'}(0)$ holds.

Each F_h in V_5 is computed by honest server h as follows:

$$F_h = \left(f_{\mathrm{P}}(h) - P'^{(h)}\right)\left(\sum_{h' \in H} f_{\mathrm{R}_{h'}}(h) + \sum_{c \in C} R_c^{(h)}\right) + \sum_{h' \in H} f_{0_{h'}}(h) + \sum_{c \in C} Z_c^{(h)} + f_{\mathrm{D}}(h). \quad \text{(A.1)}$$

Defining

$$\Delta_h = f_{\mathrm{P}}(h) - P'^{(h)} = f_{\mathrm{P}}(h) - f_{\mathrm{P}'}(h),$$

$$f_{\mathrm{R}}(x) = \sum_{h\in H} f_{\mathrm{R}_h}(x) = \sum_{i=0}^{t} \rho_i x^i,$$

$$f_{\mathrm{Z}}(x) = \sum_{h\in H} f_{0_h}(x) = \sum_{i=1}^{2t} z_i x^i,$$

Eq. (A.1) is written as

$$F_h = \Delta_h \left(f_{\mathrm{R}}(h) + \sum_{c\in C} R_c^{(h)} \right) + f_{\mathrm{Z}}(h) + \sum_{c\in C} Z_c^{(h)} + f_{\mathrm{D}}(h).$$

Then, all values in V_3, V_4, V_5 are represented by the following linear equation:

$$\begin{pmatrix} \sum_{h\in H} f_{\mathrm{R}_h}(c_1) \\ \vdots \\ \sum_{h\in H} f_{\mathrm{R}_h}(c_t) \\ F_{h_1} \\ \vdots \\ F_{h_{t+1}} \\ \sum_{h\in H} f_{0_h}(c_1) \\ \vdots \\ \sum_{h\in H} f_{0_h}(c_t) \end{pmatrix} = \begin{pmatrix} 0 \\ \vdots \\ 0 \\ \Delta_{h_1} \sum_{c\in C} R_c^{(h_1)} + \sum_{c\in C} Z_c^{(h_1)} + f_{\mathrm{D}}(h_1) \\ \vdots \\ \Delta_{h_{t+1}} \sum_{c\in C} R_c^{(h_{t+1})} + \sum_{c\in C} Z_c^{(h_{t+1})} + f_{\mathrm{D}}(h_{t+1}) \\ 0 \\ \vdots \\ 0 \end{pmatrix} + M \begin{pmatrix} \rho_0 \\ \vdots \\ \rho_t \\ z_1 \\ \vdots \\ z_{2t} \end{pmatrix}, \qquad \text{(A.2)}$$

where

$$M = \begin{pmatrix} A & 0 \\ E & K \\ 0 & B \end{pmatrix},$$

and

$$A = \begin{pmatrix} 1 & c_1 & c_1^2 & \cdots & c_1^t \\ \vdots & \vdots & \vdots & \ddots & \vdots \\ 1 & c_t & c_t^2 & \cdots & c_t^t \end{pmatrix}, B = \begin{pmatrix} c_1 & c_1^2 & \cdots & c_1^{2t} \\ \vdots & \vdots & \ddots & \vdots \\ c_t & c_t^2 & \cdots & c_t^{2t} \end{pmatrix},$$

$$E = \begin{pmatrix} \Delta_{h_1} & \Delta_{h_1} h_1 & \Delta_{h_1} h_1^2 & \cdots & \Delta_{h_1} h_1^t \\ \vdots & \vdots & \vdots & \ddots & \vdots \\ \Delta_{h_{t+1}} & \Delta_{h_{t+1}} h_{t+1} & \Delta_{h_{t+1}} h_{t+1}^2 & \cdots & \Delta_{h_{t+1}} h_{t+1}^t \end{pmatrix}, K = \begin{pmatrix} h_1 & h_1^2 & \cdots & h_1^{2t} \\ \vdots & \vdots & \ddots & \vdots \\ h_{t+1} & h_{t+1}^2 & \cdots & h_{t+1}^{2t} \end{pmatrix}.$$

Because all values $(\rho_0, \dots, \rho_t, z_1, \dots, z_{2t})$ in the last term of Eq. (A.2) are chosen by the honest servers, they have uniform distribution. Thus, if the matrix M is nonsingular, then (V_3, V_4, V_5) as a whole has uniform distribution and is independent from $\Delta_h, f_{\mathrm{D}}, R_c^{(h)}, Z_c^{(h)}$, and the choice of C. The non- singularity of M is evaluated as follows.

Let E_i be the i-th row of the matrix E and K_i' be the matrix obtained by removing the i-th row from the matrix K. Then

$$\det M = \sum_{i=1}^{t+1} \left((-1)^{i-1} \det \begin{bmatrix} A \\ E_i \end{bmatrix} \cdot \det \begin{bmatrix} K_i' \\ B \end{bmatrix} \right),$$

$$\det\begin{bmatrix} A \\ E_i \end{bmatrix} = \Delta_{h_i} \det\begin{bmatrix} & A & & \\ 1 & h_i & \cdots & h_i^t \end{bmatrix} = (-1)^t \Delta_{h_i} \left(\prod_{c\in C}(c-h_i)\right)\left(\prod_{\substack{c'>c \\ c,c'\in C}}(c'-c)\right),$$

and

$$\det\begin{bmatrix} K_i' \\ B \end{bmatrix} = \left(\prod_{h\in H\backslash\{h_i\}} h\right)\left(\prod_{c\in C} c\right)\left(\prod_{h\in H\backslash\{h_i\}}\left(\prod_{c\in C}(c-h)\right)\right)$$
$$\times\left(\prod_{\substack{c'>c \\ c,c'\in C}}(c'-c)\right)\left(\prod_{\substack{h'>h \\ h,h'\in H\backslash\{h_i\}}}(h'-h)\right).$$

By noting

$$f_{\mathrm{P}}(0) - f_{\mathrm{P}'}(0) = \sum_{h_i\in H} \Delta_{h_i}\left(\prod_{h\in H\backslash\{h_i\}} h\right)\left(\prod_{h\in H\backslash\{h_i\}}(h-h_i)\right)^{-1},$$

we obtain

$$\det M = (-1)^t \left(\prod_{c\in C} c\right)\left(\prod_{\substack{c'>c \\ c,c'\in C}}(c'-c)\right)^2 \left(\prod_{h\in H}\left(\prod_{c\in C}(c-h)\right)\right)$$
$$\times\left(\prod_{\substack{h'>h \\ h,h'\in H}}(h'-h)\right)\left(f_{\mathrm{P}}(0) - f_{\mathrm{P}'}(0)\right).$$

Hence, $\det M \neq 0$ holds if and only if $f_{\mathrm{P}}(0) = P \neq f_{\mathrm{P}'}(0)$. Note that polynomial $f_{\mathrm{P}'}(x)$ is determined by the values $f_{\mathrm{P}'}(h) = P'^{(h)}$ sent from the attacker to the honest servers as password's shares. So, $f_{\mathrm{P}'}(0)$ can be considered as a "guessed" password P'. Consequently, (V_3, V_4, V_5) has no additional information if the guessed password P' is not equal to the registered password P. This means that, no matter how the attacker acts co-operating with the corrupted servers, it cannot get additional information beyond the on-line dictionary attack.

In this attack model, the attacker is assumed to corrupt the t servers in L. If one thinks this assumption not realistic, we can slightly modify the scheme as follows. For every possible set L, **Pre-computation and communication phase** is performed by the servers in L (rather than all servers). When L is specified by the data owner in **Data reconstruction phase**, servers use shares of random numbers and "0" for the specified L. The used shares must be discarded, but shares for other set $L'(\neq L)$ need not to be discarded. The security of this modified protocol is proven in a similar

way to the above proof. Note that if the number of servers n is $2t+1$, we need not modify the scheme.
